# Optimization of Electrochemical Sensitivity in Anticancer Drug Quantification through ZnS@CNS Nanosheets: Synthesis via Accelerated Sonochemical Methodology

**DOI:** 10.1016/j.ultsonch.2024.106858

**Published:** 2024-03-24

**Authors:** Pin-Yi Chen, T. Keerthi Reddy, Umamaheswari Rajaji, Asma A. Alothman, Mani Govindasamy

**Affiliations:** a Department of Mechanical Engineering, Ming Chi University of Technology, New Taipei City 24301, Taiwan; b International Ph.D. Program in Innovative Technology of Biomedical Engineering and Medical Devices, Ming Chi University of Technology, New Taipei City 24301, Taiwan; c Department of Mechanical Engineering, Chang Gung University, Taoyuan City 33302, Taiwan; d Centre for Applied Research, Saveetha School of Engineering, Saveetha Institute of Medical and Technical Sciences (SIMATS), Chennai 602105, Tamil Nadu, India; e Research Center for Intelligence Medical Devices, Ming Chi University of Technology, New Taipei City 243303, Taiwan; f Department of Chemistry, College of Science, King Saud University, Riyadh 11451, Saudi Arabia

**Keywords:** Electrochemical methods, Anticancer drug, Graphitic carbon nitride, Zinc sulfide, Binary nanosheets, Sonochemical synthesis

## Abstract

Zinc sulfide/graphitic Carbon Nitride binary nanosheets were synthesized by using a novel sonochemical pathway with high electrocatalytic ability. The as- obtained samples were characterized by various analytical methods such as Transmission Electron Microscopy (TEM), Field emission scanning electron microscopy (FESEM), Energy-dispersive X-ray spectroscopy (EDS), X-ray diffraction analysis (XRD), and X-ray photoelectron spectroscopy (XPS) to evaluate the properties of ZnS@CNS synthesized by this new route. Subsequently, the electrical and electrochemical performance of the proposed electrodes were characterized by using EIS and CV to establish an electroactive ability of the nanocomposites. The complete properties like structural and physical of ZnS@CNS were analyzed. As-prepared binary nanocomposite was applied towards the detection of anticancer drug (flutamide) by various electrochemical methods such as cyclic voltammetry (CV), differential pulse voltammetry (DPV) and amperometry. The glassy carbon electrode modified with a ZnS@CNS composite demonstrates a remarkable electrocatalytic efficiency for detecting flutamide in a pH 7.0 (PBS). The composite modified electrode shows synergistic effect of ZnS and CNS catalyst. The electrochemical sensing performance of the linear range was improved significantly due to high electroactive sites and rapid electron transport pathways. Crucially, the electrochemical method was successfully demonstrated in biological fluids which reveals its potential real-time applicability in the analysis of drug.

## Highlights

• Innovative and sonochemical synthesis of ZnS@CNS composite without any toxic reducing agent.
• The morphological, spectral, and electrochemical characterization of the ZnS@CNS composite platform was carried out.
• ZnS@CNS composite/GCE platforms are exhibits excellent electrochemical properties for anticancer drug detection.
• This sensor was applied for real time test in the biological fluids.

## Graphical abstract

**Figure:**
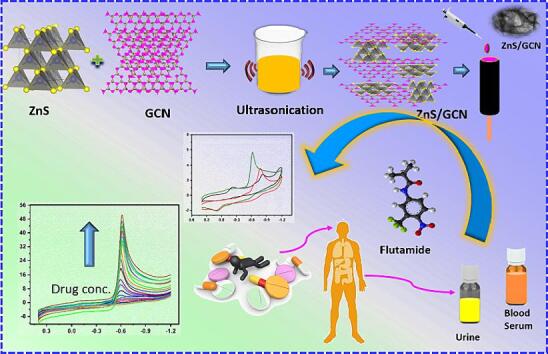

## 1 Introduction

**Scheme 1:**
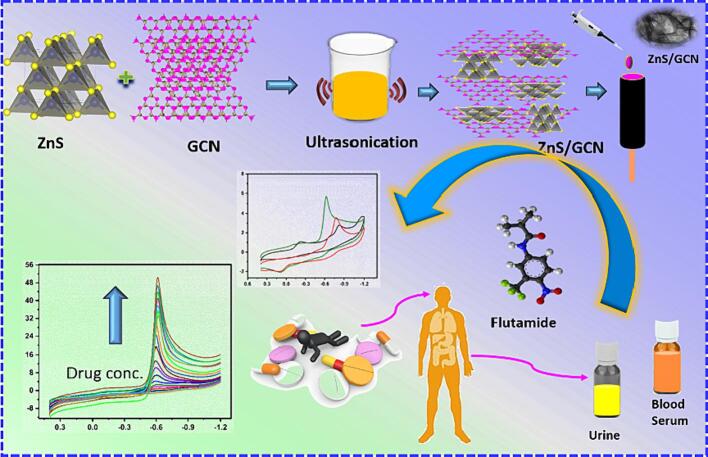
Facile Synthesis of ZnS Supported Carbon Nitride for Detection of Chemotherapeutic Drug in biological fluids.

Flutamide is an anti-cancer drug which is known as a non-steroidal anti-androgen. Testosterone, a naturally occurring hormone found in males, plays a crucial role in the growth and spreading of prostate cancer cells in men at humans [1]. It is a well-known synthetic, anti-androgen, non-steroidal drug which is useful for treating people who are suffering from prostate cancer [2]. On the other hand, for women who are suffering from poly-cystic ovarian syndrome (PCOS) is suggested to take flutamide for controlling the over secretion of testosterone. Flutamide is broadly used for the treatment of prostate cancer because of its best metabolic activity, good efficacy and oral intake procedures [3]. Flutamide is taken in the form of tablet, which is 250 mg a day for 3 times to limit the testosterone by prostate cancer cells [4]. Flutamide was chemically organized as (2- methyl -N-[-nitro-3-(trifluoromethyl)phenyl]-propanamide). Flutamide works on the blocking of the effects of natural male hormone testosterone, which results in decrement of growing and spreading of prostate cancer cells [4], [5]. Due to high dosage of flutamide in humans which causes the side effects in human. They are methemoglobinemia, loss of sexual interest/ability, blood in urine, vomiting, rectal bleeding, enlarged growth of male breasts, drowsiness, diarrhea, liver malfunction, hot flashes [6]. In detection of drugs and developing with electrochemical sensor is quite demanding because the complications of the drugs in biological system and environmental resources. Pharmaceutical product development is the most important thing for controlling and management of various diseases. Mainly, prostate cancer is one of the major issues in the biological system [7]. Hence, constructing a rapid and sensitive technology for detection of flutamide which is present in human blood, and urine samples. More importantly, nanomolar detection is our main goal for determining the level in biological fluids. As a result, we got an opportunity for developing an efficient technique for optimal detection of flutamide which enhances the reduction of the related health risk simultaneously (Scheme 1).

Electrochemical methods are a very well-known and convenient path to approach the preparation of coatings with concentration of the electrolyte and monomers, electrodeposition time, and considered thickness by controlling the voltage [8], [9]. These methods can be removed and recovered by any dense metals which is based on the principle of metals that can be precipitated in their elemental form on to the electrodes when their electricity or potential is applied on to the electrode [10]. All electrochemical methods are based on the interaction of electrical energy and matter. The detection of flutamide has been generally done by traditional method of analysis, such as high performance thin-layer liquid chromatography (HPLC) [11], mass spectrophotometry [1], fluorescence quenching, spectrofluorimetric determination, first flow-injection method, reverse phase high performance liquid chromatography (RP-HPLC) and electrochemical methods [12]. As these methods have complex procedures, expensive instrumentation, their poor repeatability, limited to some materials and the necessary of skillful handling make them more vulnerable in terms of practical uses. The electrochemical biosensors have high selectivity and sensitivity [[13], [14]]. The temperature, storage conditions and acidity are the specified working conditions of these biosensors which have an overall activity and a direct impact on stability of the enzyme. Therefore, we can develop enzyme-less electrochemical sensors where they are sensitive with short analysis time portable, selective, and cost-effective. It was very easy to conduct the experiment. They are often specific to a particular oxidation state of an element. They have high sensitivity, selectivity, low detection limits and possibility of real time results. The advantage of them is that the signal is electrical but there will be no transformation of required information to control units.

Sonochemistry is a chemical and ultrasound system under the working of ultrasonic radiation and its chemical reactions. The ultrasonic method involves breaking down large nanoparticles into smaller ones by subjecting them to rapid heating and cooling rates [15]. Sonochemical synthesis offers notable benefits, such as its quick quenching rate and operation under ambient conditions, along with the advantages of simplicity, energy efficiency and ultrasound-based synthesis are green technology without toxic chemicals and high temperature. Ultrasonication in the synthesis process has significant impacts, including improved mixing and dispersion of reactants, disruption and reassembly of micelles, expedited hydrolysis and condensation reactions, as well as controlled pore size and morphology. These effects contribute to a more even distribution of micelles and enhanced structural order. Ultrasonic techniques can notably enhance the mass transfer of electrolyte ions and activate the surface of materials [16]. In comparison to the traditional synthesis, and preparation methods for binary nanosheets, ultrasonication expedites the intercalation of intercalating ions into the layer spacing of graphitic carbon nitride and facilitates the subsequent exfoliation with zinc sulfide sheets in the sonochemical system. The rapid exfoliation can lead to the formation of a bi-layer nanocomposite with stacking defects and point vacancies. These binary nanosheets also exhibit elevated efficacy in electrocatalysis for electrochemical reactions, extending their utility in biosensor applications.

Zinc sulfide (ZnS) stands as a crucial II-VI semiconductor material distinguished by a wide band energy gap of approximately 3.37 eV which is useful in many application fields like lithium-sulfur batteries, photocatalytic degradation [17], photocatalytic hydrogen evolution [18], and various applications [19]. It is a low-price metal sulfide, and it shows very good catalytic behavior, outstanding biocompatibility, and chemical stability. Therefore, it can be widely used in the fields of sensors and various electronic devices [[20], [21]]. ZnS is an important component of photocatalytic material in hydrogen production. It has advantages like uniform distribution, stable structure, protection from environment and non-toxicity [22]. It has enhanced electrocatalytic property because of its high redox chemistry and crystalline structure. For preparation of zinc-based nanomaterials there are many methods like thermal evaporation and chemical vapor deposition but widely using method is hydrothermal method because of its simplicity and high efficiency.

Carbon nitrides are compounds and matrix, where there is only the combination of carbon and nitrogen atoms. Carbon nitrides are typical polymeric metal free semiconductors and electrocatalytic material [23]. Carbon nitrides material properties are controlled according to their structure and crystalline properties. Carbon nitrides are the most stable allotrope under all the ambient conditions. Carbon nitride is a 2D polymeric sheets like structure of catalyst. Its structure is constructed by tri-s-triazine units which are connected by nitrogen atoms. Carbon nitrides are monolayers in structure. The interlayer distance for carbon nitrides is 3.19 Å. The lattice distance for carbon nitrides is 7.14 Å. These materials are prepared by calcination method. The carbon nitride when coupled with electro-chemical signal which strengths the substrate because of its electrode kinetics, enriched catalytic activity, high electrochemical active surface area on the electrode. Carbon nitrides are useful for tribological coatings, chemically inert coatings, biocompatible, insulators, medical coatings and for energy storage solutions.

## 2 Experimental part

### 2.1 Materials and methods

Zinc acetate, sodium sulfide nonahydrate, choline chloride, fructose, and all other chemicals employed in this study were procured from Sigma-Aldrich in Seoul, South Korea, and were utilized without purification. For electrochemical characterization, we utilized potassium chloride (KCl, ACS reagent, ≥ 99 %) as a supporting electrolyte, and potassium ferricyanide (K_3_[Fe(CN)_6_], ACS reagent, ≥ 99 %) and potassium ferrocyanide (K_4_[Fe(CN)_6_], ACS reagent, ≥ 98.5 %) as redox probes. A commonly used phosphate buffer solution (PBS) was prepared by mixing appropriate proportions of Na_2_HPO_4_ (ACS reagent, ≥ 99 %) and NaH_2_PO_4_ (ACS reagent, ≥ 98 %) solutions, and pH ranges adjustments were made by using sulfuric acid (0.1 M) and sodium hydroxide (0.1 M).

The analysis of the surface characteristics of the synthesized material involved several techniques. Scanning Electron Microscopy (SEM) using the Hitachi S4700, Energy Dispersive X-ray (EDX) spectroscopy with the HORIBA EMAX XACT instrument, and Raman spectroscopy utilizing the WITech CRM2000 confocal microscopy Raman system with a 488 nm laser were employed. For phase identification, X-ray Diffraction (XRD) was conducted using the Rigaku D/maxB, DMX-2200 instrument. AC impedance spectroscopy was carried out using the Ω-Metrohm Autolab (AUT51770, 100–240 V ∼ 75VA50/60 Hz), and electrochemical measurements, including the amperometric method, were performed using the CHI 6171D Electrocatalytic Workstation in three-electrode cells. The working, reference, and counter electrodes in the electrochemical measurements were a modified Glassy Carbon Electrode (GCE) with a surface area of 0.071 cm^2^, saturated Ag/AgCl, and Pt wire, respectively.

### 2.2 Sonochemical synthesis of ZnS sheets

**Scheme 2:**
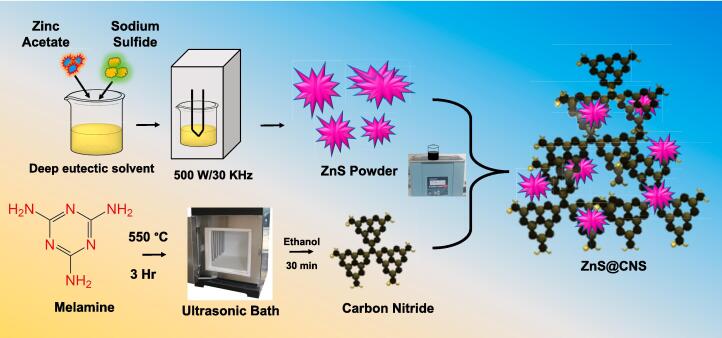
Synthesis of ZnS@CNS via sonochemical approach.

ZnS nanosheets were produced through a single-step green sonochemical method (Eq. (1)). In a typical procedure, 0.1 mol of zinc acetate [Zn(CH_3_COO)_2_] and 0.1 mol of Na_2_S were dispersed in 50 mL of a deep eutectic solvent. The resulting mixed suspension was then sonicated using an ultrasonic cleaner (with an acoustic power of 500 W and a frequency of 30 KHz) for 2 h at room temperature (25℃). The main objective of our experiment was to create nano-layers of ZnS. Subsequently, the synthesized ZnS nanolayers, appearing as white powder, were recovered through centrifugation, washed for repeated times, and then finally dried under vacuum (Scheme 2).

Overall Reaction:

(1) $$\mathrm{Zn}{\left(CH_{3}COO\right)}_{2}.H_{2}O+Na_{2}S.9H_{2}O\to ZnS+\;2CH_{3}COONa+10H_{2}O$$

### 2.3 Synthesis of CNS

Carbon nitride sheets (CNS) were synthesized via a thermal method. Briefly, melamine was finely grinded using a mortar and pestle. The resulting finely grinded powder was then transferred to an alumina crucible, covered with a lid, and heated to 550 °C for 3 h at a rate of 5 °C/min. Subsequently, the mixture was permitted to cool to room temperature, and then subjected to washing with ultrapure water and ethanol. The resulting CNS powder is further finely grinded after that it is sonicated in an ultrasonic bath with ethanol for 30 min, and further it was used for electrochemical sensing applications.

### 2.4 Sonochemical preparation of ZnS@CNS

In a typical synthesis, 5 mg/mL of CNS is prepared in ethanol, and 10 mg/mL of zinc sulfide is added, and the entire mixture is stirred for 30 min. Finally, the ZnS@CNS nanocomposite is separated through freeze-dried.

### 2.5 Fabrication of modified electrode

In a typical synthesis procedure, a solution of 5 mg/mL of carbon nitride sheets (CNS) in ethanol was prepared, and 10 mg/mL of zinc sulfide is added. The complete mixture is kept under stirring for a duration of 30 min. Ultimately, the resulting ZnS@CNS nanocomposite was separated through freeze-drying.

## 3 Results and discussion

### 3.1 Chemical and physical properties of ZnS@CNS

**Fig. 1:**
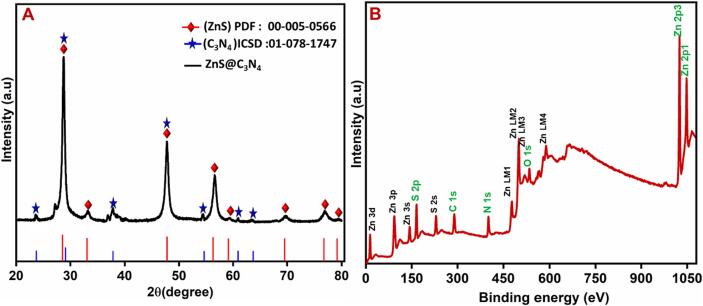
(A) XRD analysis of the Composite. (B), XPS analysis of composite.

Fig. 1 represents the XRD spectra of ZnS and ZnS@CNS. The XRD spectra of the ZnS display major diffraction peaks at 2θ values are 28.1**˚**, 32.5**˚**, 48.2**˚**, 56.1**˚**, 59.2**˚**, 69.4**˚**, 77.4**˚** and 79.1**˚** degree with corresponding to the diffraction planes (1 1 1), (2 0 0), (2 2 0), (3 1 1), (2 2 2), (4 0 0), (3 3 1), and (4 2 0) respectively [24]. The identified diffraction peaks were matched to the cubic crystal structure of the ZnS nanomaterials by using the (PDF:00–005-0566) [24]. The XRD spectra of ZnS@CNS displays all the peaks of ZnS nanomaterial in addition to CNS peaks were assigned at 26.9**˚** in the plane (0 0 2) represents the formation of existing of ZnS@CNS in absence of any other additional impurities [[25], [26]]. From Debye-Scherrer formula, we have calculated the average crystal size of the synthesized compound.

(2) $$d=\frac{0.89\lambda }{\beta Cos\theta }$$

Where, d is the Crystallite size, λ represents the Wavelength of the X-ray, β signifies the Full Width at half maximum (FWHM) of the highest intensity peak of 2θ, 0.89 was the Scherrer's constant, θ refers the Bragg diffraction angle respectively. The average particle size of ZnS, determined through the full width at half maximum (FWHM) of the (1 1 0) crystal plane, was found to be 255 nm. By comparing crystal sizes, it is suggested that the crystallinity of ZnS@CNS is superior to that of ZnS.

**Fig. 2:**
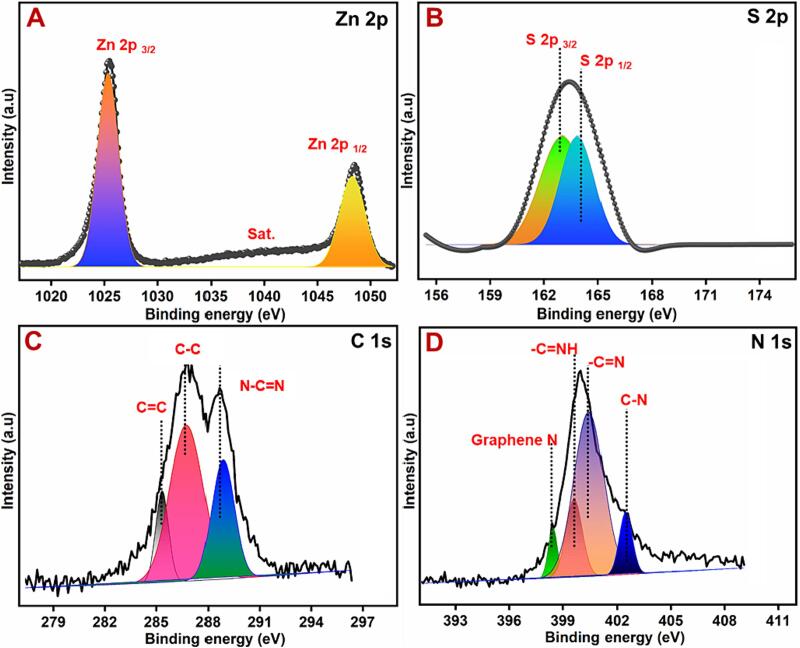
XPS analysis of the composite and elemental analysis of Zn2p, S2p, C1s, and N1s.

X-ray photoelectron spectroscopy (XPS) was examined to further investigate the chemical bonding and states of the ZnS/CNS composites. XPS analysis of the ZnS/CNS catalyst was studied, and it given in the Fig. 1B as a survey spectrum the composite. In addition, the composite of the individual elements was studied in the Fig. 2A-D. Based on the XPS analysis of Fig. 2, confirm the elements and oxidation state of the elements such as Zn 2p (Zn^2+^), S 2p (2^-^), C 1 s (4^+^) and N 1 s (3^-^) [27]. The peak separation of the peaks at 1022.5 eV and 1045.9 eV in Zn 2p spectrum (Fig. 2A) and which was originated from the Zn 2p_3/2_ and Zn 2p_1/2_ of ZnS [[27], [28]]. In other hand, the S 2p could be deconvoluted into the peaks at 162.5 eV and 163.2 eV, assigned to S 2p_3/2_ and S 2p_1/2_ of ZnS (Fig. 2B) [[27], [29]].

**Fig. 3:**
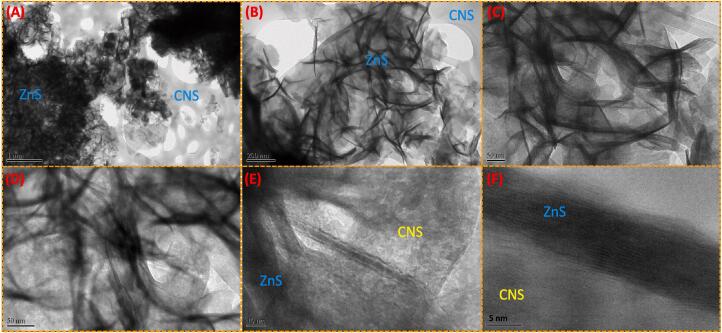
(A-F) TEM images of ZnS@CNS nanomaterial.

The XPS analysis of the C 1 s in Fig. 3B, three main peaks are observed with the binding energies at 285.2 eV, 286.5 eV and 288.9 eV can be found on the C 1 s core-level spectrum of C_3_N_4_ nanosheets, which are assigned to sp^2^ C = C bonds of graphitic carbon, C-C sp^3^-coordinated carbon bonds and sp^2^-bonded carbon (N-C = N) of the s-triazine rings, respectively [[26], [29]]. The N 1 s signal of C_3_N_4_ nanosheets also shows three feature peaks, corresponding to the sp^2^-bonded N(C-N = C) (397.8 eV), tertiary nitrogen N-(C)_3_ groups (399.4 eV), amino groups (C-N-H) (400.9 eV) and C = N group (402.5 eV) [26]. From the XPS analysis and the peaks relating to the groups of carbon nitride nanosheets. Therefore, we concluded that the composite formation based on the XPS analysis via sonochemical synthesis.

### 3.2 Morphological and elemental analysis of ZnS@CNS

The transmission electron microscopy (TEM) generates huge signals at the surfaces of the solid particle by using high-beam electrons. The crystalline structure, components, texture, and orientations of the samples were understanding by the TEM monographs. We have examined the morphological analysis of the ZnS material by TEM method (Fig. 3). Microwave and sonochemical synthesized of ZnS appears a sheets like nanoparticles in Fig. 3. TEM images of ZnS@CNS, ZnS particles are decorated with CNS sheets in Fig. 3 and CNS is layered nanosheets.

### 3.3 Electrochemical characteristics analysis

**Fig. 4:**
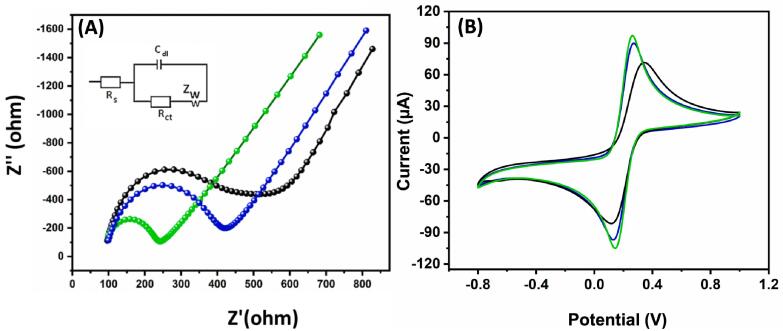
(A) EIS analysis and (B) CV analysis of ZnS/GCE, CNS/GCE (blue), and ZnS@CNS/GCE (green) in 5 mM [Fe(CN)_6_)]^3−/4−^ having 0.1 M KCl.

The electrochemical performance of electrodes and modified electrodes is predominantly linked to the reaction mechanism that governs electron and charge mobility [[30], [31]]. For better understanding the behaviour of electrode and electrolyte at the interface was done through EIS analysis. The Nyquist plot of ZnS/GCE, CNS/GCE, and ZnS@CNS/GCE in 5 mM [Fe(CN)_6_)]^3−/4−^ having 0.1 M KCl is shown in Fig. 4**A**. Further, the modified electrode was confirmed because of the various R_ct_ values. The ability towards sensing different materials represents the low charge transfer resistance (152.4 Ω) and semi-circular nature for ZnS@CNS/GCE.

The CV analysis of ZnS/GCE, CNS/GCE, and ZnS@CNS/GCE in 5 mM [Fe(CN)_6_)]^3−/4−^ having 0.1 M KCl is shown in Fig. 4**B.** ZnS and CNS modified electrode is shows a good current responses. Mainly, Epa/Epc is very low for CNS/GCE compared to ZnS/GCE. In other hand, the composite modified GCE shows excellent current performance and Epa/Epc is also very low. It means, the ZnS@CNS/GCE have a high electrocatalytic ability and these kinds of materials are suitable for electrochemical sensors and biosensors applications.

### 3.4 Electrochemical detection of flutamide via CV method

**Fig. 5:**
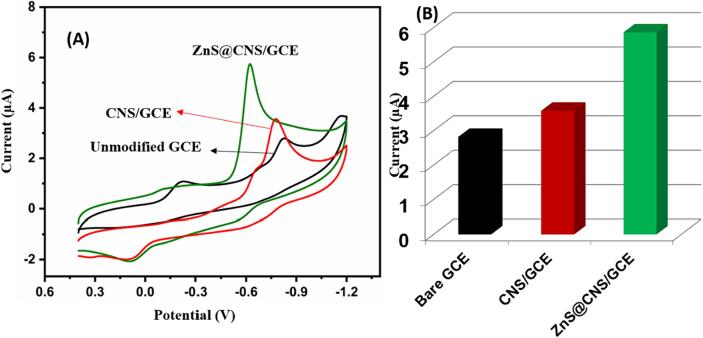
(A) CV analysis of bare GCE (black), CNS/GCE (brown) and ZnS@CNS/GCE (green) containing 75 µM of flutamide in 0.05 M PBS at 50 mV/s. (B), corresponding calibration plot. (For interpretation of the references to colour in this figure legend, the reader is referred to the web version of this article.)

**Scheme 3:**
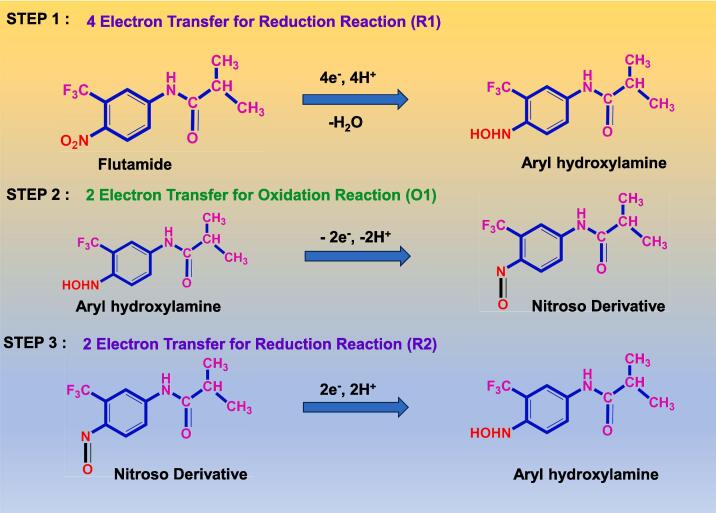
Electrochemical mechanism of flutamide detection based on ZnS@CNS/GCE.

Fig. 5A depicts the CV curves of unmodified GCE, CNS /GCE, and ZnS@CNS/GCE in presence of 75 μM of flutamide in 0.05 M PBS (pH 7.0) at the sweep rate of 50 mV/s. The bare GCE exhibits low response to flutamide, indicating that there is an inefficiency in sensing the model drug. The presence of CNS/GCE results in smaller oxidation and reduction peaks for flutamide, signifying a limited sensing capability (Fig. 5B). Conversely, ZnS@CNS/GCE demonstrates an effective reduction of flutamide to amine hydroxyl with a 4H^+^/4e^-^ system, evident from sharp irreversible cathodic peak at −0.62 V. Additionally, an oxidation peak for flutamide is observed at 0.12 V, and a small reduction peak was observed at −0.24 V, representing a 2H^+^/2e^-^ quasi-irreversible reaction attributed to the conversion of amine hydroxyl to a nitroso group (Scheme 3). The −0.62 V reduction peak is selected for further evaluation due to its sharp and higher peak current compared to others.

**Fig. 6:**
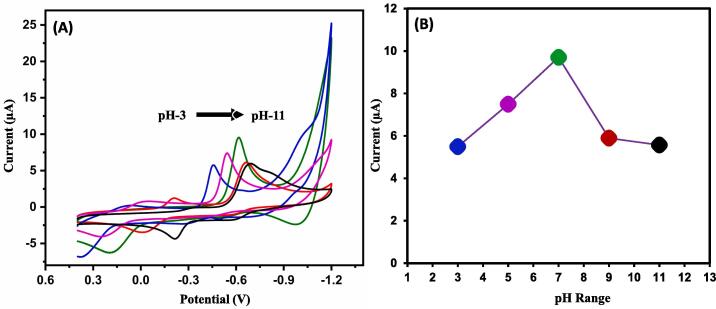
(A) CV response of flutamide at various pH ranging from 3.0 to 11 in 0.05 M PBS at ZnS@CNS/GCE and pH vs current response (B).

Here, one crucial factor in electrochemical detection lies in the medium of the pH where the reaction takes place with analyte. Therefore, we have evaluated the electrochemical detection of flutamide in existence of ZnS@CNS/GCE with different pH. The CVs of flutamide with pH from 3 to 11 is given in Fig. 6A. Here we have observed that there is a linear reduction current from pH 3 to 7 and then there is a decline from pH 9 to 11. There is a shift towards negative potential and less response of current at the basic pH. This can be due to availability of more number of hydroxyl ions and low number of H^+^ ions at basic pH and it shows the involvement of protons in the electrochemical reduction of flutamide. As, pH 7 was a physiological pH of humans and it was examined as optimum pH which was preferential pH (Fig. 6B).

**Fig. 7:**
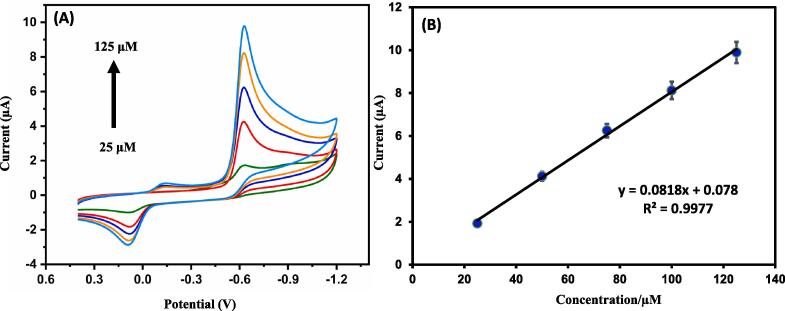
(A) CV obtained from various concentration of flutamide from 25 to 125 μM in 0.05 M PBS (pH 7.0) at a sweep rate of 50 mV/s for ZnS@CNS/GCE and (B) the corresponding linear calibration plot for cathodic current vs. [flutamide]/μM.

For evaluating the effect of flutamide on its reduction in existence of ZnS@CNS/GCE was done through various concentrations of flutamide ranging from 25 µM to 125 µM (Fig. 7A). As we are increasing the concentration, the current response is also increasing, and it reaches a maximum response of current at around 25 for 125 μM concentration of flutamide. This is because of formation of large reduction product and increased ionic stability in the electrolyte which is shown in Fig. 7B. Here, we represented the good linearity with (R^2^) value of 0.9977 corresponding to the calibration curve and which was given in Fig. 7B.

**Fig. 8:**
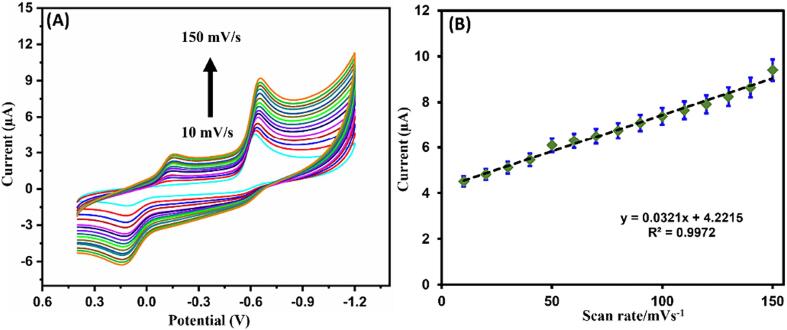
(A) Cyclic voltametric curves of flutamide at various sweep rates between 10 and 150 mV/s in 0.05 M PBS (pH 7.0) by using ZnS@CNS/GCE. (B) Linear plot of the diverse sweep rates (mV/s) vs Current (µA).

Scan rate was varied and examined through the CV of flutamide in existence of ZnS@CNS/GCE. The CVs of flutamide with various scan rate with equal interval of scan rate (10 mV/s to 150 mV/s) was shown in Fig. 8A. Here by increasing the scan rate the curve look linear with R^2^ = 0.9972 was shown in Fig. 8B, which represents the surface-controlled reduction of flutamide which depends upon ZnS@CNS/GCE.

### 3.5 Electrocatalytic reduction of flutamide at ZnS@CNS/GCE by LSV method

**Fig. 9:**
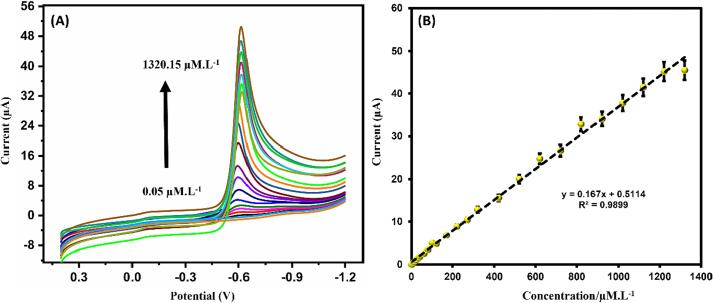
(A) LSV analysis of flutamide in 50 mM PBS (in pH 7.0) using ZnS@CNS/GCE and (B) calibration plot for cathodic current vs. [flutamide]/μM.

LSV is a sensitive and suitable voltametric technique for the determination of flutamide. Here the LSV curves of flutamide under optimized conditions were shown at different concentration of flutamide ranging from 0.05 to 1320.15 µM. L^-1^ and depicts in Fig. 9A. Here we have observed the increase of current with respect to increase of [flutamide] with good linearity. Here, the associated calibration curve illustrates a wide linear range from 0.05 to 1320.15 µM/L, which is followed by the regression equation y = -0.167x −0.5114 and an R^2^ value of 0.9899 shown in Fig. 9B. Here the modified sensor demonstrates a calculated sensitivity of 2.352 µA µM^−1^ cm^−2^. The LOD (Limit of detection) was determined utilizing formula 1, yielding a calculated value of 12.6 nM for this modified sensor. Our modified sensor performance is compared with previous articles in Table 1.

(3) LOD=3S/b

**Table 1:** Performance of sensor was evaluated with various articles.

**Electrode**	**LOD** **(µM)**	**Range** **(µM)**	**Sensitivity** **(µA/µM.cm^2^)**	**Method**	**Ref.**
Gold electrode	1.8	6–600	–	DPV	[32]
FeVO_4_-150	0.054	0.06–777.46	1.4	DPV	[33]
GO/GCE	0.006	0.009–1.9	29.55	LSV	[2]
(CB/β-CD)	0.016	0.05–158.3	5.476	DPV	[34]
(AuNP@rGO/PPy	0.003	0.001–46.11	50.06	DPV	[35]
(ZnMn_2_O_4_-PGO)	0.008	0.05–3.5	1.05	DPV	[36]
nano-Ag/MGCE	9.33	100–1000	–	DPV	[37]
MoS_2_-CZO	0.005	0.019–668.5	0.35	DPV	[38]
SF-CTAB	0.007	0.016–658.51	–	DPV	[39]
β-Cu_2_V_2_O_7_/PC	0.062	0.01–2.11	24.33	AMP	[40]
Sn-ZnO/rGO	0.073	0.01–170	14.10	DPV	[41]
g-C_3_N_4_/GCE	0.05	2–1208	1.166	DPV	[42]
Pencil graphite	0.016	0.398–6.36	–	LSV	[43]
**ZnS@C_3_N_4_**	**12.6 nM**	**0.05**–**1320.15**	**2.352**	**LSV**	**Current**

Where, S-slope and b-intercept of the plot. Here we achieved a low Limit of Detection (LOD), a wide dynamic linear range, and notable sensitivity with the ZnS@CNS/GCE is superior and compared with the electrochemical detection of flutamide with many other reported methods that was depicted in Table 1. Here, the ZnS@CNS/GCE modified electrode exhibits favourable electrochemical performance and demonstrates ability in the reduction towards the detection of anti-cancer drugs under pH 7.0 conditions.

GO = Graphene oxide, CB = carbon-black, CD = cyclodextrin, PPy = polypyrrole, rGO = reduced Graphene oxide, PGO = porous reduced graphene oxide, CZO = cerium-zirconium oxide, SF = Strontium ferrite nanocrystals, CTAB = Cetyltrimethylammonium bromide, PC = porous carbon, DPV = Differential pulse voltammetry, LSV = Linear Sweep Voltammetry, AMP = Amperometry.

The interference studies are more important in the electrochemical sensors. We studied the interference properties with common interfering species such as drugs and other biological chemicals using ZnS@CNS/GCE through LSV technique. Here by using LSV technique with PBS of pH 7.0 with N_2_ purged in 100 µM of flutamide and we are examining this through LSV. Here we have studied and added the selectivity studies, 5-folds high volume of drugs (dopamine, uric acid, folic acid, tamitinol, Adriamycin) and biological chemicals (acetylcholine, adenine, cysteine, estradiol, guanine). Here it shows the good selectivity of ZnS@CNS/GCE towards the detection of flutamide in presence of biological analytes and drug with no considerable change in detection of flutamide (<7.5 % deviation). The another most important feature in electrochemical sensor is stability of the modified electrode. To evaluate the stability of ZnS@CNS/GCE we have used 100 µM of flutamide by using LSV technique for 30 days. The modified electrode ZnS@CNS/GCE exhibited excellent stability, retaining 95.4 % of its original current even after a 30-day period, indicating its excellent stability and durable performance. Here we have fabricated six different electrodes for detecting the 100 µM of flutamide in N_2_ purged PBS of pH 7.0 to find reproducibility of ZnS@CNS/GCE by using LSV technique. Here we got the very good reproducibility of the ZnS@CNS/GCE through the RSD of LSV peaks is less and found to be 1.26 %. We have examined the repeatability of ZnS@CNS by using the flutamide (100 μM) through LSV technique for 10 times. The ZnS@CNS/GCE demonstrated superior repeatability, as evidenced by the lower Relative Standard Deviation (RSD) of 1.24 %.

The practical utility of modified electrodes relies on their effectiveness in detecting the analyte in biological samples. Here by using LSV method we are performing the electrochemical sensing of ZnS@CNS/GCE towards detection of flutamide in serum and urine samples. By utilizing our prior experimental knowledge, specified concentration of flutamide drug is dissolved in fluids. Subsequently, we have taken 4 various concentrations (20, 50, 100, and 200 µM) of added volume for the analysis by using the Linear Sweep Voltammetry (LSV) technique in a PBS with ZnS@CNS/GCE, through the standard addition real sample method. The recovery analysis was found to be in the range of 96.3 % to 99.2 % (Table 2). The outcomes signify exceptional performance in the analysis of real samples.

**Table 2:** Real sample analysis in various biological fluids by using the ZnS@CNS/GCE.

**Real samples**	**Added** **(nM)**	**Obtained (nM)**	**Recovery (%)**	**RSD** **(%)**
Serum	0	–	–	–
	20	19.4	97.0	1.67
	50	48.9	97.8	2.04
	100	96.9	96.9	2.74
	200	197.2	98.6	1.84
Urine	0	–	–	–
	20	19.26	96.3	1.97
	50	49.15	98.3	1.84
	100	98.92	98.92	1.09
	200	198.4	99.2	1.43

## 4 Conclusion

In current work, CNS decorated ZnS NPs was developed by green microwave and sonochemical approach. The successful decoration of ZnS with CNS sheets was observed in various characterization results. Here, we have constructed a non-enzymatic sensor for determination of drugs in biological fluids. Here flutamide which is an anti-prostate cancer, and it was analysed based on modified GCE. The improved electrochemical performance observed in ZnS@CNS/GCE can be because of synergistic properties of ZnS (surface activity) and CNS sheets (conductivity and surface area). The modified electrode exhibits a wide linear range from 0.05 to 1320.15 µM for flutamide, with the capability to detect nano-molar concentrations (12.6 nM) by using LSV analysis. The sensitivity of the sensor is evaluated as 2.352 µAµM^-1^cm^−2^. The practical applicability in pharmaceutical industries was spoken by the recovery results of real sample analysis. Improved sensitivity, low limit of detection (LOD), and reliable reproducibility and repeatability collectively demonstrate the effectiveness of ZnS@CNS/GCE in the non-enzymatic detection of flutamide. Compared to previous articles and reports, our modified sensor shows high performance towards detection flutamide based ZnS modified CNS composite.

## CRediT authorship contribution statement

**Pin-Yi Chen:** Conceptualization, Funding acquisition, Investigation, Supervision. **T. Keerthi Reddy:** Data curation, Investigation, Writing – original draft. **Umamaheswari Rajaji:** Methodology, Writing – review & editing. **Asma A. Alothman:** Conceptualization, Formal analysis. **Mani Govindasamy:** Writing – review & editing, Project administration, Resources.

## Declaration of competing interest

The authors declare that they have no known competing financial interests or personal relationships that could have appeared to influence the work reported in this paper.
